# African Biochemists Plan More Collaboration

**DOI:** 10.1100/tsw.2000.16

**Published:** 2001-04-04

**Authors:** Peter N. Campbell

## Abstract

The Federation of European Biochemical Societies (FEBS) was the first regional organisation of biochemists, holding its first congress in London in 1964. There followed the creation of the Pan American Association of Biochemical Societies (PAABS) and then the Federation of Asian and Oceanian Biochemists (FAOB). An obvious development was the formation of a similar organisation to take care of Africa, but this proved impossible so long as apartheid survived in South Africa. With the removal of the latter, the way was clear for the foundation of the Federation of African Societies of Biochemistry and Molecular Biology (FASBMB). The first congress of the new federation was held in Nairobi in September 1996 under the Presidency of Prof. Dominic Makawiti of Nairobi University. Among the 300 participants were representatives from 19 countries in Africa. The second congress was held at Potchefstroom in South Africa in 1998 and the third was just held in Cairo.

The Regional organisations are linked to the International Union of Biochemistry and Molecular Biology (IUBMB) as Associate Members and each, except for FEBS, derives some income from the Union for various activities including support of congresses, education workshops and other meetings.

While from the outset the FASBMB elected an excellent Executive Committee, it would be true to say that its true potential has not so far been achieved. At the Council meeting held in Cairo, Prof. Vincent Titanji of Cameroon was elected President with Prof. Ali Khalifa of Egypt as Vice President and Prof. Meissa Toure of Senegal as Meetings Councellor. Prof. Iqbal Parker of South Africa continues as Secretary General and Dr. Julia Hasler of Zimbabwe continues as Treasurer. The Council resolved to redouble the efforts of the Federation to effectively enhance collaboration among African biochemists. In this respect the new methods of electronic communication are surely crucial (e.g., INASP, supported by the International Council for Science, has set up AJOL 2000 to offer free access to the contents of all scholarly journals published in Africa). A Web site is being created and a series of newsletters are planned following the launch of the first excellent one by Dr Ignatious Ncube of Zimbabwe. Further activities of the Education Committee under Dr. Trevor Anderson (South Africa) are planned, as are courses on particular techniques such as the one on protein structure currently being organised by Prof. Titanji in Cameroon. While funds from the IUBMB are much appreciated, other sources of income will have to be pursued if the full potential of the Federation is to be achieved.

Without doubt it will take determination and courage to pursue the objectives of the federation in the circumstances often present in Africa. Weather conditions, drought or floods create chaos; universities are shut following violent demonstrations; AIDS and devastates all classes of society. But surely there has never been a time when it is more important to encourage the development of a cadre of experts in the potential benefits of biochemistry and molecular biology for the resolution of health and agricultural problems. In brief, the problems must be overcome and the scientists must demonstrate to the politicians that the citizens of the various countries can work together to make the best use of their limited resources. Egypt to the North and South Africa to the South have particularly important roles to play as leaders since their economies are in better shape than that of many other counties but the Africans also need the help of like-minded biochemists in the industrial world. My message to African biochemists is that many international organisations want to help but they must be assured that their support is going to be effectively used. I believe that the new Executive Committee of the FASBMB is able and willing to respond appropriately.

Peter N. Campbell, PhD.
December 2000

Previously posted in NewsLab, TheScientificWorld.
© 2000 with author.
